# Development of an ultrasound guided focused ultrasound system for 3D volumetric low energy nanodroplet-mediated histotripsy

**DOI:** 10.1038/s41598-022-25129-x

**Published:** 2022-11-30

**Authors:** Bar Glickstein, Ramona Aronovich, Yi Feng, Tali Ilovitsh

**Affiliations:** 1 Department of Biomedical Engineering, Tel Aviv University, Tel Aviv, Israel; 2 The Key Laboratory of Biomedical Information Engineering of Ministry of Education, Department of Biomedical Engineering, School of Life Science and Technology, Xi’an Jiaotong University, Xi’an, People’s Republic of China; 3 The Sagol School of Neuroscience, Tel Aviv University, Tel Aviv, Israel

**Keywords:** Biomedical engineering, Mechanical engineering

## Abstract

Low pressure histotripsy is likely to facilitate current treatments that require extremely high pressures. An ultrasound guided focused ultrasound system was designed to accommodate a rotating imaging transducer within a low frequency therapeutic transducer that operates at a center frequency of 105 kHz. The implementation of this integrated system provides real-time therapeutic and volumetric imaging functions, that are used here for low-cost, low-energy 3D volumetric ultrasound histotripsy using nanodroplets. A two-step approach for low pressure histotripsy is implemented with this dual-array. Vaporization of nanodroplets into gaseous microbubbles was performed via the 1D rotating imaging probe. The therapeutic transducer is then used to detonate the vaporized nanodroplets and trigger potent mechanical effects in the surrounding tissue. Rotating the imaging transducer creates a circular vaporized nanodroplet shape which generates a round lesion upon detonation. This contrasts with the elongated lesion formed when using a standard 1D imaging transducer for nanodroplet activation. Optimization experiments show that maximal nanodroplet activation can be achieved with a 2-cycle excitation pulse at a center frequency of 3.5 MHz, and a peak negative pressure of 3.4 MPa (a mechanical index of 1.84). Vaporized nanodroplet detonation was achieved by applying a low frequency treatment at a center frequency of 105 kHz and mechanical index of 0.9. In ex-vivo samples, the rotated nanodroplet activation method yielded the largest lesion area, with a mean of 4.7 ± 0.5 mm^2^, and a rounded shape. In comparison, standard fixed transducer nanodroplet activation resulted in an average lesion area of 2.6 ± 0.4 mm^2^, and an elongated shape. This hybrid system enables to achieve volumetric low energy histotripsy, and thus facilitates the creation of precise, large-volume mechanical lesions in tissues, while reducing the pressure threshold required for standard histotripsy by over an order of magnitude.

## Introduction

Cavitation-based histotripsy is a focused ultrasound (US) technology used for noninvasive tissue ablation^1^. This method employs short (milliseconds), high ultrasonic bursts to fractionate tissues mechanically^2^. When it is targeted within bulk tissue, histotripsy produces a dense bubble cloud that collapses rapidly, producing a shock wave that can cause injury to the tissue^3^. Studies have shown that histotripsy has great potential for the treatment of cancer^4–6^, thrombolysis^7^ and kidney stones^8^. However, the extremely high peak negative pressures (PNP) (around 20 MPa^9^) required for the generation of a cavitation nuclei constitute one of its key drawbacks since the US pulses need to be precisely focused on the target area to avoid cavitation in healthy neighboring cells. Boiling histotripsy is another technique that uses lower pressures and longer pulses (milliseconds) to generate boiling bubbles to liquefy target tissue. Yet, these pressures are still around ~ 10 MPa^10^. To reduce the high pressure, histotripsy can be combined with microbubbles (MBs) or nanodroplets (NDs)^5,11–13^. NDs are liquid droplets, with diameters ranging from 100 to 750 nm. Their small size facilitates their penetration into capillaries, and enhances their extravasate into tumor tissue^14^. Upon US insonation, the NDs can vaporize into MBs^15–17^.

In this paper, we developed an ultrasound guided focused ultrasound (USgFUS) system for low-cost 3D volumetric histotripsy using NDs. This setup is composed of a rotating imaging transducer that it located within a low frequency therapeutic transducer that operates at a center frequency of 105 kHz. The rotating imaging transducer was used for volumetric ND activation into gaseous MBs which are then detonated by the low frequency therapeutic transducer to trigger potent mechanical effects in the surrounding tissues. The pressure threshold required for ND activation at low frequencies is usually higher than at MHz frequencies. Therefore, the activation at center frequencies of 1 MHz and below needs mechanical index (MI) values exceeding 1.9^18^. For this reason, US imaging transducers that operate at MHz frequencies are optimal for NDs vaporization. In contrast, therapeutic US that operates at low frequencies (below 250 kHz) was shown to enhance MBs oscillation^3,19^. In order to improve treatment efficiency and safety, an integrated system of US imaging and therapy is proposed.

Two methods were previously used to integrate US imaging and therapy. The first uses two independent US imaging and therapeutic systems^20–22^. Alternatively, US imaging and therapy can be combined in a single system^21–23^. The main advantage of the combined system is that both transducers are co-aligned, which facilitate the dual imaging-therapy of a target, as they are less susceptible to target motion, do not depend on target geometry and provide higher precision. These systems use a fixed 1D imaging transducer. Since a 1D array is used to image a completed 3D anatomic volume, the obtained 2D US images are inherently incomplete. Here, by accommodating a rotating imaging transducer in a hybrid USgFUS system, low-cost 3D volumetric US imaging capabilities are made feasible, and are used for the activation of NDs.

Conventional 3D US imaging either captures multiple images by using a moving 1D transducer and stacking the individual 2D images to form a 3D image, or by using 2D matrix arrays that enable real time volumetric imaging. Nevertheless, the design and fabrication of the 2D matrix array probes still face hurdles^24^. Compared to 1D arrays, 2D matrix arrays remain very expensive and difficult to produce because of the many transducer elements. The relatively low price of US scanners is one of the factors that accounts for the widespread use of US imaging; thus, the high cost of 3D US systems curtails their usage in the clinic. Alternatively, low-cost fast volumetric US imaging can be achieved using 1D arrays associated with controlled mechanical rotation of the transducer, where the set of multiple 2D images are combined into a 3D image^25,26^. Clinical applications of 3D US imaging using either matrix arrays or a moving 1D array have been reported to be of value in cardiology^27–29^, surgical guidance^30–32^, fetal imaging^33–35^, and also 3D US localization microscopy^36^. In the context of NDs, 3D US imaging of these agents has been described^37^. Still, activation of NDs using a 1D moving transducer has not yet been investigated.

**Figure 1:**
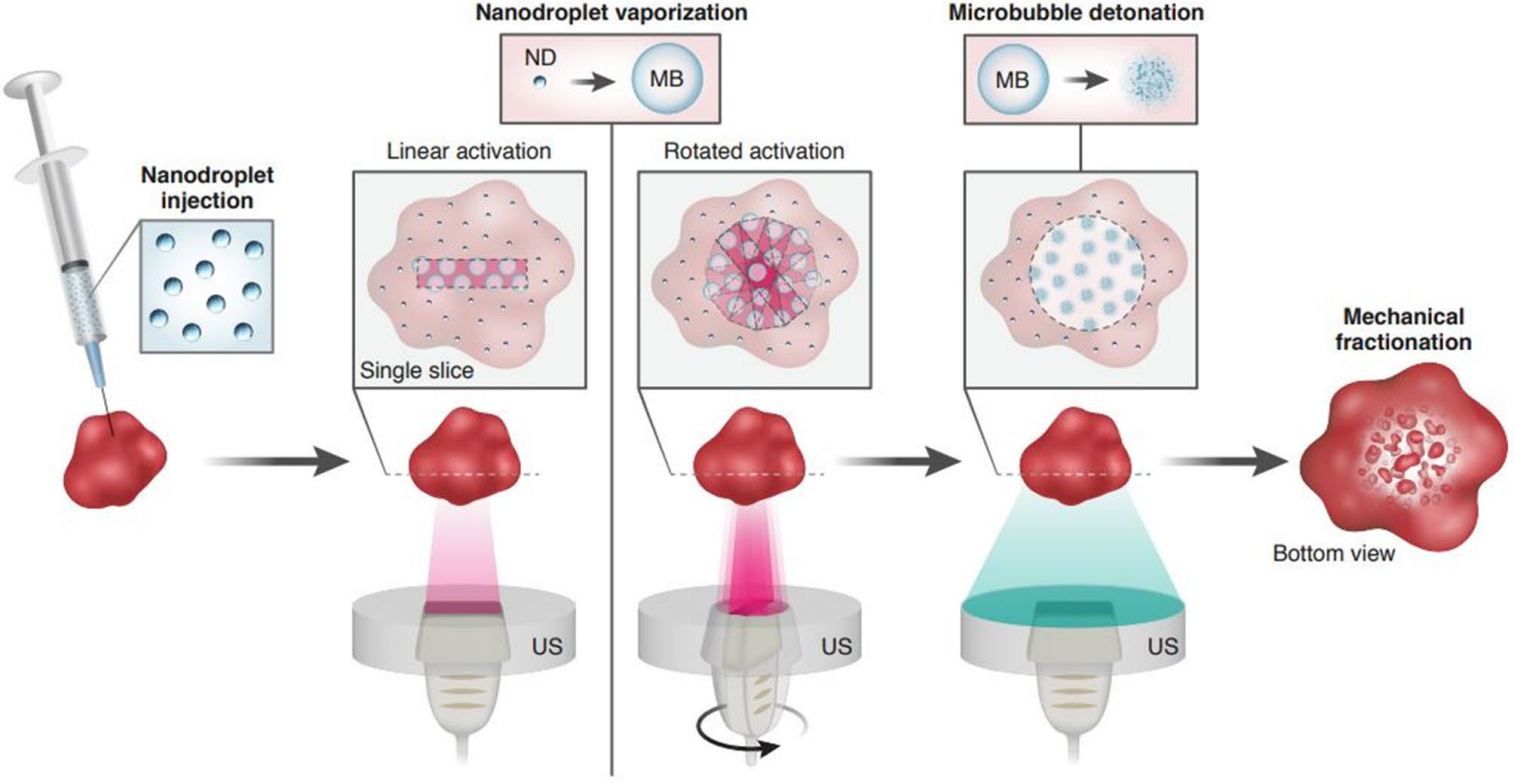
Schematic illustration of the two-step 3D volumetric ND-mediated histotripsy method. NDs are injected into the sample, followed by their vaporization into MBs. In the standard linear activation, an US beam is swept at a specific focal depth, creating a linear shape of activated NDs. When the US probe is rotated, the resulting activation shape is the superposition of the multiple rotating activation lines that creates a circular shape. This imaging transducer is situated within a therapeutic transducer that is used to detonate the vaporized NDs and create mechanical lesions in the sample.

Here, volumetric NDs activation was achieved via the 1D rotating imaging probe to yield a large circular activation area. Then, low frequency US at a center frequency of 105 kHz was applied to the vaporized NDs to detonate them and achieve significant mechanical damage at the target tissue site. Importantly, both steps were carried out while operating below an MI of 1.9, in accordance with Food and Drug Administration (FDA) guidelines and reduce the pressure threshold required for standard histotripsy by two orders of magnitude. There are two major differences compared to previous work^38^. First, a 1D fixed imaging array was previously used for ND activation. This created a linear pattern of vaporized NDs as a result of the linear sweep of the focused beam. When rotation of the probe is added, a rotating linear pattern of vaporized ND is created, yielding a volumetric circular shape at the focal depth. By detonating the vaporized NDs, a round-like shape is formed (Fig. 1). In addition, previously, two separate US imaging and therapy systems were used. The fixed 1D imaging array was placed perpendicularly to the therapeutic transducer. This results in complex mechanical alignment of the two focal spots and poses geometrical constrains on the treated object. The system developed here utilizes co-aligned transducers, with low-cost 3D imaging capabilities that makes it possible to treat larger volumes while operating under low pressures.

## Results

### Theoretical prediction of microbubble expansion

**Figure 2:**
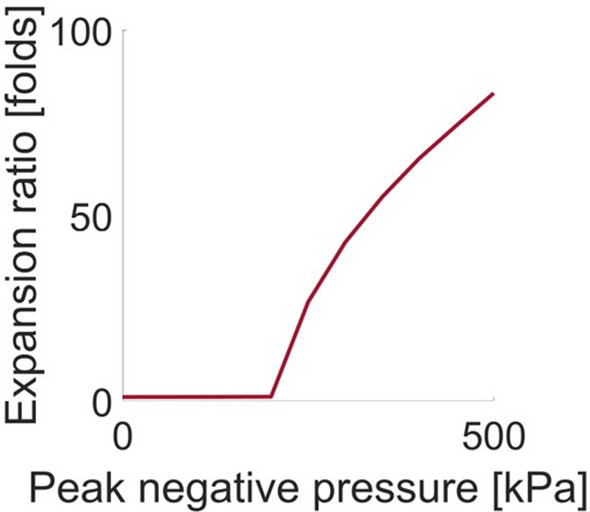
Theoretical predictions of the MB maximal expansion ratio as a function of the PNP at a center frequency of 105 kHz and for a MB initial radius of 0.75 μm.

Numerical stimulations were performed using the Marmottant model to estimate the MB expansion ratio as a function of the PNP for a center frequency of 105 kHz (Fig. 2). These predictions were used to identify the low frequency parameters that were used for the second step of vaporized NDs detonation. The fabricated NDs diameter was ~ 300 nm. Following vaporization, their predicted size is five times larger and therefore average MB diameter of 1.5 µm was simulated^17^. Previous work showed that the MB transition to inertial cavitation occurs at expansion ratios above 3.5^3^, hence assessing the MB expansion ratio can indicate the range of PNPs required for the process. When working with an MI of 0.9 (PNP of 290 kPa), the predicted expansion ratio was ~ 38 compared to an expansion ratio of ~ 3.5 and ~ 1 when working with an MI of 0.6 (PNP of 200 kPa) and 0.4 (PNP of 130 kPa), respectively.

### Nanodroplet vaporization optimization

**Figure 3:**
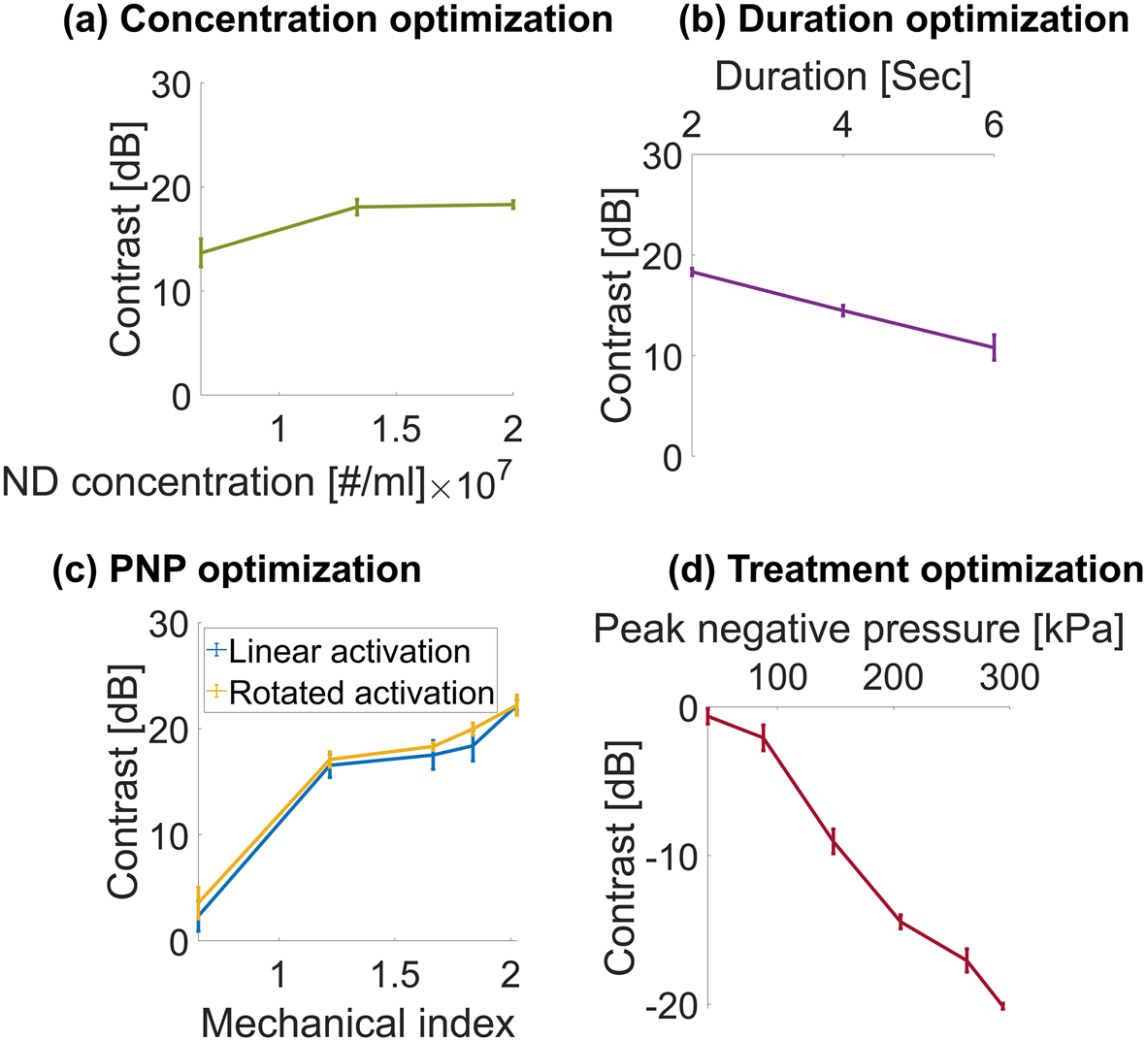
ND vaporization and detonation optimization results. Contrast inclusion before and after ND vaporization as a function of (**a**) ND concentration; (**b**) Activation duration; and (**c**) Activation pulse mechanical index. (**d**) Contrast reduction as a function of PNP for vaporized NDs at a center frequency of 105 kHz. All experiments were performed in triplicate. All data are plotted as the mean ± SD.

The activation process was optimized using the USgFUS system setup. NDs with a mean diameter of 300 nm were fabricated. Upon activation, vaporized ND diameter is expected to grow by a factor of ~ 5 and have an average diameter of 1.5 µm^17^. The NDs were vaporized with the 1D rotating imaging transducer at a center frequency of 3.5 MHz that yielded volumetric vaporization. The activation concentration (Fig. 3a), the activation duration (Fig. 3b) and the applied PNP (Fig. 3c) were optimized. US images were acquired before ND vaporization, where the inclusion appeared dark, and after ND vaporization, where the inclusion became hyperechoic as a result of MB generation. The contrast difference was calculated and analyzed as a function of each parameter. The contrast for ND concentrations of 1.32 × 10^7^ NDs/mL and 2 × 10^7^ NDs/mL was similar (Fig. 3a); hence, a concentration of 2 × 10^7^ NDs/mL was chosen and used in all subsequent experiments. This experiment was conducted without applying transducer rotation. Next, rotated activation using multiple rotation speeds which translated into different activation durations were tested. An activation duration of 2 s yielded the highest contrast of 18.3 dB and was used for the subsequent experiments (Fig. 3b). A comparison of standard linear activation and rotated activation using different MI showed similar behavior. The contrast increased when increasing the activation pulse MI (Fig. 3c); thus, in order to maximize the contrast while operating below an MI of 1.9, we chose to work with an MI of 1.84, which yielded a contrast of 20 ± 0.6 dB for rotated activation and 18.4 ± 1.4 dB for linear activation.

### Low frequency insonation optimization

This experiment was performed to confirm the numerical simulations by assessing the reduction in contrast as a function of the applied PNP upon low frequency insonation (Fig. 3d). Following activation of NDs into MBs, low frequency US excitation at a center frequency of 105 kHz was applied to the MB-filled inclusion. US images were acquired before and after treatment. When inertial cavitation occurs, the MB collapses, so that there should be less contrast as the PNP increases and more MBs collapse, as can be seen in Fig. 3d.

### Ex-vivo ablation results

**Figure 4:**
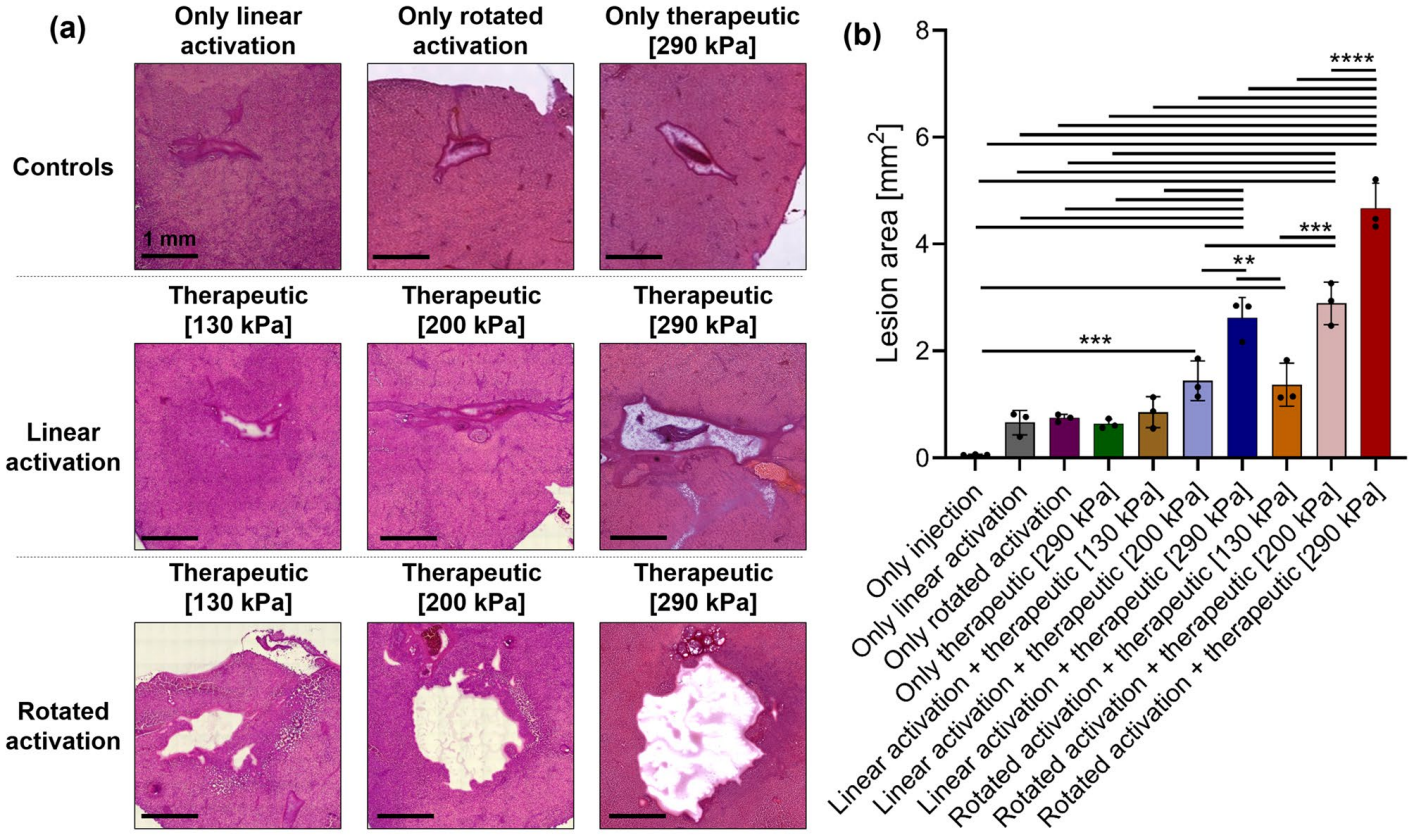
ND-mediated low energy histotripsy evaluation in ex-vivo samples. (**a**) Histological photomicrographs of ND+ only linear activation control, ND+ only rotated activation control, ND+ only therapeutic control, ND-mediated histotripsy at a center frequency of 105 kHz and MI of 0.4, 0.6 and 0.9 with linear ND activation and rotated ND activation. (**b**) Quantification of the lesion area for each group. All experiments were performed in triplicate. Adjusted p values were **p < 0.01, ***p < 0.001, and ****p < 0.0001. All data are plotted as the mean ± SD.

To evaluate the differences between volumetric histotripsy using rotated activation and standard histotripsy using linear activation and to assess the morphology of each method, this approach was implemented in ex-vivo chicken liver samples. NDs activation into MBs was performed using the optimized parameters while applying low frequency US at a center frequency of 105 kHz at PNPs of 130 kPa (MI = 0.4), 200 kPa (MI = 0.6), or 290 kPa (MI = 0.9) to the vaporized NDs for each of the treated groups. The mechanical damage was then subjected to histological evaluation (Fig. 4a). For the standard approach, the lesion was linear in shape, since the beam is swept at a specific focal distance. By contrast, for the rotated activation, the lesion was more circular in shape as a result of the rotation of the transducer. The lesion dimensions were quantified by analyzing the histology images (Fig. 4b). The lesion area increased as a function of MI. The mean lesion areas for the volumetric activation were 1.4 ± 0.3 mm^2^, 2.9 ± 0.3 mm^2^, and 4.7 ± 0.5 mm^2^ for MI values of 0.4, 0.6, and 0.9, respectively. In comparison to lesion area of 0.85 ± 0.2 mm^2^, 1.4 ± 0.3 mm^2^, and 2.6 ± 0.4 mm^2^ for MI values of 0.4, 0.6, and 0.9, respectively, when using the fixed 1D transducer (p < 0.0001). The ‘only injection’, ‘only linear activation’, ‘only rotated activation’ and ‘only therapeutic [290]’ controls had lesion areas of 0.06 ± 0.007 mm^2^, 0.66 ± 0.19 mm^2^, 0.74 ± 0.11 mm^2^ and 0.63 ± 0.35 mm^2^, respectively.

## Discussion

Histotripsy is a widely investigated method for noninvasive US surgery which has taken great strides forward in recent years^39–42^. The combination of NDs and histotripsy has been proposed for targeted tumor treatment since these NDs with their small diameters can extravasate from blood vessels into the tumor tissue^5,16,18,43,44^. To overcome the challenges that result from the high energy needed to carry out histotripsy, a method for ND-mediated histotripsy was developed using a two-step approach^38^. The aim of this study was to develop a new platform for low energy volumetric US histotripsy. The method uses a uniquely designed hybrid USgFUS system which contains a rotating imaging array at a center frequency of 3.5 MHz for ND activation set inside a therapeutic transducer operating at 105 kHz for MB detonation. The ability to rotate an imaging transducer precisely, with predefined speed and acceleration, while it is aligned to the rotation axis at the center of a therapeutic transducer, cannot be performed with a hand-held probe that is being manually rotated. By precisely rotating the imaging transducer with a controlled motor, volumetric activation of the NDs is achieved. This results in a circular-shaped lesion that makes it possible to treat larger volumes simultaneously. By controlling the width of the scan line and the focal depth, the treated volume can be adjusted. However, the treated volume is still limited by the depth of field of the focal spot of the therapeutic transducer (for the transducer used in this study it was 40 mm). Full 3D control of both foci in the USgFUS system could be achieved using a multi-element therapeutic transducer, instead of the single element transducer that was used here. This will facilitate the co-alignment of both the activation and low frequency foci. Here the NDs were directly injected into the tissue. Since their location was well defined, they could not be pushed using the imaging or therapy probes. In a tumor model, the NDs are expected to be confined within the tumor region in a similar manner. The main goal of this study was to validate the two-step approach on the new USgFUS setup, optimize the parameters, and provide a proof of concept that rotated activation of NDs can further maximize the generated mechanical damage.

ND vaporization experiments were used to evaluate the effects of the ND concentration, activation duration and MI on contrast. The main advantage of the rotational probe is the creation of a circular lesion, compared to a linear elongated shape for the fixed imaging transducer. This effect cannot be directly seen in the contrast experiments, where the NDs were already confined within a rod-shape volume. Therefore, the improvement in lesion area was demonstrated in the ex vivo experiments. The contrast experiments were a perquisite step to identify the optimal activation parameters that yield the maximal contrast within the inclusion; that is, the highest vaporized ND concentration. An ND concentration of 2 × 10^7^ NDs/mL, combined with a treatment duration of 2 s and an MI of 1.84 yielded the best results and were used in the subsequent experiments. The contrast remained similar for concentrations of 2 × 10^7^ and 1.32 × 10^7^ NDs/mL (Fig. 3a). The results showed that as the treatment duration increased, the contrast decreased (Fig. 3b), suggesting that prolonged activation initiates the destruction of some of the vaporized NDs and therefore leads to a decrease in contrast. The contrast increased as the MI of the activation pulse increased for both the rotated activation and fixed activation groups (Fig. 3c). A contrast increase by 22 dB and 20 dB was achieved for MI of 2 and 1.84, respectively. An MI of 1.84 was chosen to remain below the safe MI of 1.9. A comparison of the fixed activation and rotated activation showed that rotated activation resulted in a similar contrast, as can be seen in Fig. 3c. It should be noted that the imaging was performed using the 1D array at a fixed angle. Since the cavity had a rod-shaped volume, a ND activation event, created a vaporized ND that moved freely and contributed to the contrast of the entire volume (for the linear or rotated activation). The vaporized ND generation rate depends on the ultrasound excitation parameters, the pulse repetition frequency (PRF) and the total activation duration. Here, the same activation duration and PRF were applied in the linear and rotation modes, and therefore it can be expected that the total number of vaporized NDs will be similar. Given the cavity shape, the vaporized NDs can distribute within the volume, and therefore when imaging a cross section with the imaging transducer, the contrast obtained with the linear vs. rotational activation was similar. However, this is not the case in the ex vivo samples, where the vaporized NDs were confined within the tissue.

Next, the impact of low frequency insonation of the vaporized NDs on contrast reduction as a function of the PNP was assessed (Fig. 3d). A contrast decrease of 15 dB was observed for a pressure of 200 kPa and was further reduced by over 20 dB for a pressure of 300 kPa. This is consistent with numerical stimulations showing that an expansion ratio of 3.5 occurs at a PNP of ~ 200 kPa. Therefore, above this PNP, the MB transition to inertial cavitation. The Marmottant model is typically used for shelled MBs. Here it was used to identify the low frequency therapeutic parameters that were applied to the vaporized NDs. Fluorescence microscopy studies showed that vaporized NDs can retain their shell^45^. Even if this was not the case, it was shown that at low frequencies (below 250 kHz), the oscillations of lipid-shelled MBs were similar to that of clean gas bubbles, and the effect of the MB shell was negligible^3^. Therefore, even if the vaporized bubbles do not have a shell, the Marmottant model can still be used here to accurately model the vaporized NDs behavior as a function of the PNP at the low frequency of 105 kHz. The low frequency chosen for this step was 105 kHz. Our previous study compared ND-mediated histotripsy at 850, 250 and 80 kHz, and found that 80 kHz was the most effective^38^. Therefore, here we used the closest frequency that was supported by the system (105 kHz). The numerical simulations predicted a similar expansion ratio for both 105 and 80 kHz frequencies, when operating with the same MI of 0.9. It should be noted that at frequencies below 800 kHz, it is unclear if the MI definition is valid, however we used it in the absence of an alternative safety metric for low-frequency therapeutic ultrasound. We worked with a maximal MI value of 0.9 that is well below the upper limit of 1.9. In addition, the MI is defined in the absence of contrast agents. Here, we used it to avoid collateral damage in the tissues that surround the treated area. These areas do not contain NDs, and hence the use of MI is valid for these regions. The regions that contain NDs are the treated area where mechanical damage is intended to occur.

Finally, 3D volumetric histotripsy was evaluated in ex-vivo chicken liver experiments (Fig. 4). With direct bubbles injection, we have consistently used a concentration of 2 × 10^7^ bubbles/mL (either MBs or NDs), both ex vivo and in vivo^19,38,46^, hence this was the chosen concentration. We acknowledge that the injected dose can vary between studies and also depend on the type of injection (intravenous vs. direct injection^47^). Thus, to facilitate the comparison to previous research, the same concentration was used here. To assess the extent of damage and its morphology resulting from the new approach, the fixed and rotated activation approaches were compared, in addition to fours control groups (Only ND injection, ND+ only linear activation, ND+ only rotated activation, and ND+ only low frequency therapeutic treatment). Treatment was applied for 2 min (based on previous studies^38,46^) and consisted of an activation pulse at a MI of 1.84 in parallel with low frequency excitation at a center frequency of 105 kHz and PNPs of 130 kPa (MI of 0.4), 200 kPa (MI of 0.6) and 290 kPa (MI of 0.9). A significant increase in the lesion area as a function of the MI was observed. When comparing the lesions formed for the fixed and rotated transducers, the rotated activation strategy yielded the largest lesion areas, that was more circular in shape compared to the fixed approach that yielded a smaller elongated shaped. These lesions were significantly larger than the four control groups. This suggests that rotated activation allowed the vaporized NDs to disperse over a larger area which led to greater destruction of the tissue. The 105 kHz transducer focal spot had a full-width-half-max of 10, and 40 mm in the lateral and axial axes, respectively. The lateral dimension is ~ twofold larger than the lesion size that was generated with the rotated activation approach and ~ 3.8 fold larger than the lesion size that was generated with the linear activation approach (when applying the highest PNP of 290 kHz). Thus, the differences between the mechanical disruptions were the result of the rotated activation. Importantly, the maximal lesion size here was limited by the local injection of the NDs. In future work we will use systemically injected NDs to test the generation of different sized lesions. In addition, acoustic emission monitoring during the activation and detonation steps could be included in order to monitor bubble activity^48^.

This study reports the development of a low-cost ND activation method that can be used for volumetric histotripsy. The possible future applications of this method include 3D monitoring of cavitation activity and different schemes for uniform activation of NDs. In addition, to further improve performance, full 3D activation of NDs could utilize a matrix array.

## Conclusions

A two-step method for low-cost, low-energy volumetric ND-enhanced histotripsy is presented. By precisely rotating a 1D imaging transducer within a low frequency single element transducer, a robust USgFUS system is created. Rotating the imaging transducer creates a circular vaporized ND shape, which upon detonation generates a round- shaped lesion. In comparison, an elongated lesion shape is formed when using a standard 1D imaging transducer for ND activation. Our approach facilitates the creation of large-volume mechanical damage in tissues and may be used in the future for volumetric treatment of solid tumors.

## Materials and methods

### Numerical modeling

The Marmottant model^49^ was used to predict the MB expansion ratio. The stimulation implemented MATLAB (version 2021b, Mathworks, Natick, MA). This is a popular model which was shown to have good fit with experimental observations^3,50^. The model takes parameters such as MB composition, US excitation wave and the MBs’ surrounding medium viscosity and density into consideration. Theoretical predictions were made for the MB expansion ratio as a function of the PNP (between 0 and 500 kPa) at a center frequency of 105 kHz. The parameters were identical to those in Ref.^3^. The surface tension of the MB outer radius was set to 0.073 N/m (saline) and to 0.04 N/m for the inner radius. The shell density was 1000 kg/m^3^, the shell shear modulus was 122 MPa, the shell viscosity was 2.5 Pa s, the shell surface dilatational viscosity was 7.2 × 10^9^ N, and the elastic compression modulus was 0.55 N/m. The shell thickness was set to 1.5 nm. The initial MB radius value was 0.75 μm.

### Nanodroplet preparation

The ND preparation took place in two stages. First, the MB precursor solution and activation were prepared as described previously^51,52^. Briefly, the lipids disteroylphosphatidylcholine (DSPC) and 1,2-distearoyl-sn-glycero-3-phosphoethanolamine-*N*-[meth-oxy(polyethyleneglycol)-2000] (ammonium salt) (DSPE-PEG2K) (Avanti Polar Lipids, Alabaster, AL) (1 mg per 1 mL) were combined at a molar ratio of 90:10 mol/mol and prepared using a thin film hydration method. A buffer mixture of glycerol, propylene glycol, and PBS (pH 7.4) at a volume ratio of (16:3:1) was added to the lipids and sonicated at 62 °C. The precursor solution was aliquoted into vials with a liquid volume of 1 mL and saturated with perfluorobutane. MBs were formed by standard agitation techniques using a vial shaker. In the second stage, the MBs were condensed into NDs, as described in Refs.^52,53^. The MB vials were immersed in an isopropanol bath at a temperature of − 10 °C to − 13 °C and swirled gently for approximately 2 min. A 25 G syringe needle containing 50 mL of perfluorobutane gas was then inserted into the vial septum and the plunger was depressed slowly until condensation was observed. The size distribution and concentration of the NDs were measured with a particle counter system (AccuSizer FX-Nano, Particle Sizing Systems, Entegris, MA, USA). The NDs were stored at 4 °C during the experiments. The NDs were used within 3 h of their preparation. The NDs had an average diameter of 300 nm.

### Ultrasound setup

**Figure 5:**
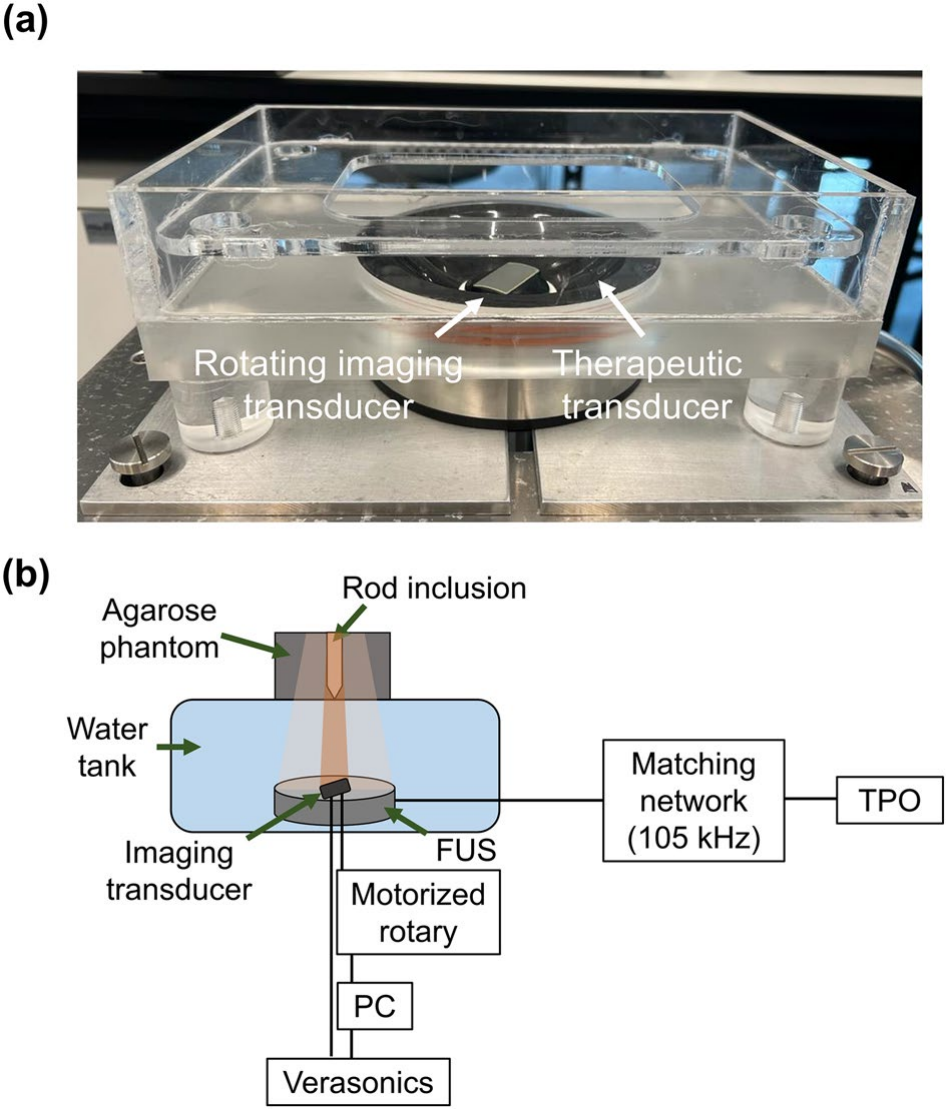
(**a**) The hybrid USgFUS system, composed of an imaging array located within a therapeutic single-element transducer. (**b**) Illustration of the experimental setup used to operate the USgFUS system.

An USgFUS was developed for the concurrent 3D activation and detonation of NDs (Fig. 5a). The system was composed of a 1D rotating imaging array controlled by a motorized rotary situated within a therapeutic transducer located at the bottom of a water tank. The focal distance of the therapeutic transducer was 65 mm; therefore, the elevation focus of the imaging transducer was selected to be located at 65 mm. Moreover, in order to facilitate deep targets imaging, an imaging transducer with a center frequency of 3.5 MHz was chosen (IP104, Sonic Concepts, Sonic Concepts, Bothell, WA, USA). This transducer has 128 elements, with an aperture elevation of 13.5 mm, and aperture azimuth of 28.2 mm. The imaging transducer was controlled by a programmable US system (Vantage 256, Verasonics Inc., Redmond, WA, USA). The imaging transducer was assembled into a motorized rotary (RTY-IP100, Sonic Concepts) composed of a rotary motor and a gear system to dynamically rotate the imaging probe precisely by ± 180° from its home position with respect to a fixed therapeutic transducer. Because this system is hermetically sealed, it provides frictionless rotation and is controlled by the user via MATLAB. The scripts provide control over the rotation angle, speed and acceleration of the attached imaging probe. The rotating imaging transducer was used for ND activation process by transmitting a 2-cycle sinusoidal pulse focused on the location of the injected NDs (at a distance of z = 65 mm). The imaging transducer was also used for acquiring US images of the NDs before and after each optimization experiment.

The therapeutic transducer was made up of a spherically focused single-element therapeutic transducer (H149, Sonic Concepts) supporting two center frequencies of 105 and 200 kHz using custom matching networks. Here, a center frequency of 105 kHz was chosen for the experiments (lateral and axial full width half maximum of 10 and 40 mm, respectively). The transducer focus was at a distance of 65 mm. The transducer was operated using a transducer power output unit (TPO-200, Sonic Concepts). Both transducers PNPs were calibrated with a needle hydrophone (NH0200, Precision Acoustics, UK) in situ. In each experiment, an agarose phantom was placed at the focal spot of both the imaging and therapeutic transducers and contained either a diluted ND solution or the ex-vivo chicken liver samples inside the rod inclusion (Fig. 5b).

### Agarose phantom preparation

Agarose phantoms were created by mixing 1.5% agarose powder (A10752, Alfa Caesar, MA, USA) and deionized water. The solution was heated until all the powder was completely dissolved, and then was poured into a custom mold and allowed to cool. The mold measured 65 mm × 25 mm × 20 mm (length × width × height) and contained an aluminum rod 15 mm in height at its center. The width of the rod was adjustable: for the optimization experiments, a mold with a 6 mm rod was used, and for the ex-vivo experiments a mold with an 8 mm rod was used. Once extracted from the mold, the phantoms were placed at the focal spots of both transducers in the USgFUS setup, and the rod-shaped cavity was filled with ND suspension.

### Nanodroplet vaporization optimization experiments

The aim of these experiments was to optimize the vaporization process of NDs into MBs and compare fixed to rotated activation. Fixed activation involves the sweeping of the US beam across a specific focal depth, unlike rotated activation where the US beam rotates, and the resulting activation shape is the superposition of multiple rotating activation lines that create a circular shape. The imaging transducer (center frequency of 3.5 MHz) was used for both ND activation and for the US image acquisition which were captured before and after the activation. The speed and acceleration used for the rotated activation were 200 degrees/s and 2000 degrees/s^2^ respectively. Activation duration, PNP and concentration were assessed. A mixture of 0.67–2 × 10^7^ NDs/mL diluted with 300 µL degassed phosphate buffered saline (PBS) was injected into the rod inclusion in the agarose mold and filled the inclusion completely. Next, a 2-cycle excitation pulse^52^, with a PRF of 20 Hz for a total duration of 2–6 s and PNPs ranging 1.2–3.8 MPa (MI of 0.65–2) was applied to the ND inclusions to vaporize the NDs into MBs. Post-processing of the captured images was used to calculate the change in contrast before and after the vaporization process, using Eq. (1)^46^:

1 $$Contrast \left[dB\right]=20{\mathrm{log}}_{10}\left(\frac{{\mu }_{i}}{{\mu }_{o}}\right),$$

where µ_i_ was the mean value of the pixels within the region inside the NDs inclusion after the activation process and µ_o_ was the mean value of the pixels of the same region before activation.

### Low frequency MB insonation optimization experiment

In these experiments, a mixture of 2 × 10^7^ NDs/mL diluted in 300 µL of degassed PBS was injected into the rod inclusion and activated into gaseous MBs. Then, a low frequency US (treatment duration of 2 s, PRF of 33 Hz and a pulse length of 0.5 ms^46^) at a center frequency of 105 kHz was applied on the vaporized NDs with PNPs ranging from 40 to 300 kPa. The US images were captured before and after low frequency application. 3D ND were vaporized via the rotated imaging probe using the optimized parameters (2 cycle sinusoid at a center frequency of 3.5 MHz, MI of 1.84, PRF of 20 Hz; total activation duration of 2 s with the beam focused to z = 65 mm). To summarize the contrast experiment was conducted as follows: 2 s of NDs activation → image capture (about 20 s) → 2 s of low frequency application → image capture. Post-processing of the captured images was used to calculate the change in contrast caused by MB destruction before and after the low frequency insonation process, using Eq. (1). Here, µ_i_ was the mean value of the pixels within the region inside the ND inclusion after the low frequency application process and µ_o_ was the mean value of the pixels in the same region before the process.

### Ex-vivo experiments

These experiments were conducted as previously described in Ref.^38^. Fresh chicken livers were cut into 15 mm × 7 mm pieces and placed within the rod inclusion inside the agar phantom. A 30 μL solution containing 2 × 10^7^ NDs in degassed PBS were injected into the center of each sample with an insulin micro syringe with a 31G needle under US imaging guidance to visualize the needle in the center of the sample prior to injection. Therefore, the NDs were located at the focal region of the transducer (z = 65 mm). The treatment duration was 120 s in which the imaging transducer activated the NDs using standard fixed activation or the rotated activation (using the optimized parameters), in parallel to the application of the low frequency treatment (PRF of 33 Hz and a pulse length of 0.5 ms) at a center frequency of 105 kHz and PNPs of 130 kPa (MI = 0.4), 200 kPa (MI = 0.60) and 290 kPa (MI = 0.9). For the rotated approach, at a given angle, the rotating imaging transducer vaporized the NDs, and the low frequency therapeutic transducer detonated the vaporized droplets. This process was repeated for all the rotation angles. In addition to the comparison between fixed and rotated activation, four control groups were included: (1) ‘ND injection only’ without any US application. (2) ‘Only linear activation’ that included NDs injection followed by activation with the fixed transducer using the optimized parameters (2 cycle sinusoid at a center frequency of 3.5 MHz, MI of 1.84, PRF of 20 Hz, total duration of 120 s). In this control, no treatment with the 105 kHz low frequency transducer was performed. (3) ‘Only rotated activation’. This control included ND injection followed by activation with the rotating transducer using the optimized parameters (2 cycle sinusoid at a center frequency of 3.5 MHz, MI of 1.84, PRF of 20 Hz, total duration of 120 s). In this control, no treatment with the 105 kHz low frequency transducer was performed. (4) ‘Only therapeutic [290 kPa]’ that included NDs injection followed by application of low frequency insonation at a center frequency of 105 kHz with the highest PNP that was used (290 kPa). Additional parameters include a PRF of 33 Hz, pulse length of 0.5 ms, and a total treatment duration of 120 s. In this control, no activation with the imaging transducer was performed. After the US treatment, all samples were flash-frozen using liquid nitrogen and methyl butan and stored at − 80 °C. The frozen samples were cryo-sectioned into 30-μm-thick slices and stained with hematoxylin (Leica 3801542) and eosin (Leica 3801602) (H&E) according to the standard procedure. The H&E slides were scanned in an Aperio Versa 200 slide scanner (Leica Biosystems, Buffalo Grove, IL) at × 20 optical magnification. For comparison and quantification of the resulting lesions, post-processing of the scanned images was performed in ImageJ. Each image was cut into a square of the same size using the same scale and magnification. The lesion area of each image was outlined such that the pixels inside the marked area turned black and the other pixels (outside the marked area) turned white. Then, the lesion area in mm^2^ was calculated as the number of black pixels multiplied by the pixel area. according to Eq. (2):

2 $$Lesion \, area \, \left[{\mathrm{mm}}^{2}\right]=\# \, black \, pixels\times pixel \, area \, \left[{\mathrm{mm}}^{2}\right].$$

### Statistics

The results are presented as the mean ± standard deviation (SD). All experiments were repeated in triplicate. The statistical analyses were conducted using Prism9 software (GraphPad Software Inc.). p values below 0.05 were considered significant.

## Data Availability

The datasets generated during and/or analyzed during the current study are available from the corresponding author upon reasonable request.
